# Bridging hypertension in children and adolescents with hypertension in adult individuals

**DOI:** 10.1186/1824-7288-41-S2-A54

**Published:** 2015-09-30

**Authors:** Gianfranco Parati, Simonetta Genovesi

**Affiliations:** 1 Department of Health Sciences, University of Milano-Bicocca, Milan, Italy; 2 Department of Cardiovascular, Neural and Metabolic Sciences, San Luca Hospital, Istituto Auxologico Italiano, Milan, 20149, Italy

## 

There is growing awareness that in children also “essential” hypertension is the most prevalent form of blood pressure (BP) elevation, and that it may be associated with target organ damage. Evidence is also available that suggests that a child with hypertension has a higher probability of being hypertensive at an adult age than a child with normal BP levels. This suggests that pathophysiological mechanisms contributing to the development of hypertension in childhood might contribute to consolidate such a condition at an adult age. As in adults, in children being overweight or obese have become an epidemic problem associated with elevated BP. This is also the case for lack of physical exercise and excessive intake of sodium with food. It has to be emphasized that in children it is not only weight excess that is associated with increased BP, but the distribution of adipose tissue also plays a role. Among young subjects with similar body weight, those with a greater waist circumference are at a greater risk of having high BP, possibly due to humoral factors released by visceral fat. Indeed, increased insulin resistance (assessed through the HOMA index), increased leptin and uric acid levels are positively and independently associated with increased BP values in children and adolescents, while an inverse correlation has been found with plasma adiponectin levels. Another factor which might contribute to hypertension in childhood is a dysfunction of autonomic neural cardiovascular modulation, characterized by an increase in cardiovascular sympathetic and a reduction in cardiac vagal modulation, documented by a reduction of cardiac arterial baroreflex sensitivity, remaining significant even after adjustment for increased body weight. These considerations underscore the importance of using techniques for 24h ambulatory BP and heart rate monitoring in the diagnostic and therapeutic management of children and adolescents with hypertension aimed at assessing their hemodynamic reactivity and the related pattern of cardiovascular autonomic nervous modulation, in daily life conditions.

Given the young age of the subjects being addressed, it is possible that pathophysiological factors contributing to hypertension might still be modifiable and that no structural cardiovascular changes have yet occurred in this age range, the appearance of which might make hypertension an irreversible condition. The hypertensive child therefore represents a challenge to the medical community for implementing interventions able to achieve true early cardiovascular disease prevention, besides being a potential model for a better understanding of the pathophysiological mechanisms underlying essential hypertension in adults.

